# Malaria prevention practices and delivery outcome: a cross sectional study of pregnant women attending a tertiary hospital in northeastern Nigeria

**DOI:** 10.1186/s12936-016-1363-x

**Published:** 2016-06-18

**Authors:** Hamzat U. Muhammad, Fatima J. Giwa, Adebola T. Olayinka, Shakir M. Balogun, IkeOluwapo Ajayi, Olufemi Ajumobi, Patrick Nguku

**Affiliations:** Nigeria Field Epidemiology and Laboratory Training Programme, Abuja, Nigeria; Department of Medical Microbiology, Ahmadu Bello University Teaching Hospital Zaria, Zaria, Nigeria; Department of Epidemiology and Medical Statistics, Faculty of Public Health, University of Ibadan, Ibadan, Nigeria; National Malaria Elimination Programme, Federal Ministry of Health, Abuja, Nigeria

**Keywords:** Malaria parasitaemia, Parturient mothers, Intermittent preventive treatment, Low birth weight, Anaemia

## Abstract

**Background:**

Malaria in pregnancy remains a public health problem in Nigeria. It causes maternal anaemia and adversely affects birth outcome leading to low birth weight, abortions and still births. Nigeria has made great strides in addressing the prevention and control of malaria in pregnancy. However, recent demographic survey shows wide disparities in malaria control activities across the geopolitical zones. This situation has been compounded by the political unrest and population displacement especially in the Northeastern zone leaving a significant proportion of pregnant women at risk of diseases, including malaria. The use of malaria preventive measures during pregnancy and the risk of malaria parasitaemia, anaemia and low birth weight babies were assessed among parturient women in an insurgent area.

**Methods:**

A cross-sectional survey was conducted among 184 parturient women at Federal Medical Centre, Nguru in Yobe state, between July and November 2014. Information on demographics, antenatal care and prevention practices was collected using an interviewer-administered questionnaire. Maternal peripheral and the cord blood samples were screened for malaria parasitaemia by microscopy of Giemsa-stained blood films. The presence of anaemia was also determined by microhaemocrit method using the peripheral blood samples. Data was analysed using descriptive and analytical statistics.

**Results:**

Prevalence of malaria parasitaemia, anaemia and low birth weight babies was 40.0, 41.0 and 37.0 %, respectively, and mothers aged younger than 25 years were mostly affected. Eighty (43.0 %) of the women received up to two doses of sulfadoxine-pyrimethamine for intermittent preventive treatment (IPTp-SP) during pregnancy and most, 63 (83.0 %) of those tested malaria positive received less than these. Presence of malaria infection at antenatal clinic enrollment (OR: 6.6; 95 % CI: 3.4–13.0), non-adherence to direct observation therapy for administration of IPTp-SP (OR: 4.6; 95 % CI: 2.2–9.5) and receiving <two doses of IPTp-SP (OR: 3.1; 95 % CI: 1.5–6.7) were significant risk factors for malaria parasitaemia at delivery.

**Conclusion:**

The high prevalence of malaria in pregnancy and the adverse outcome in this insurgence area reflects the poor access of pregnant women to preventive measures such as IPTp-SP. Effort to reach displaced pregnant women and supervision of delivery of malaria preventive measures by healthcare providers should be intensified.

## Background

In Nigeria, malaria remains a public health problem and accounts for 11 % of maternal mortality [1]. Malaria in pregnancy may cause anaemia, low birth weight (LBW) and fetal loss [1]. Nigeria adopted the World Health Organization (WHO) strategic framework for the prevention and control of malaria during pregnancy which recommends a three-pronged approach comprising intermittent preventive treatment during pregnancy with sulfadoxine-pyrimethamine (IPTp-SP), use of long-lasting insecticidal nets (LLINs), and case management of malaria illness and anaemia [2]. The 2013 National Demographic Health Survey (NDHS) reported the percentage of women who slept under insecticide-treated nets (ITNs) as 13.2 and 18.7 % in the northeast geopolitical zone and Yobe State, respectively [1]. Also it was reported that, access to IPTp-SP among pregnant women during antenatal care visit was 24.0 % in the zone, while it was only 16.7 % in Yobe State [1]. The pregnant women who took two and three doses of IPTp-SP were 16.7 and 4.5 % in the zone and only 7.4 and 3.7 % was recorded for Yobe state [1]. The current situation of political unrest, acts of terrorism, population displacement and collapse of health services in the geopolitical zone have left a significant number of pregnant women with limited access to antenatal health services. The aim of this study was to determine the use of malaria preventive strategies during pregnancy and the presence of malaria infection, anaemia and low birth weight babies at delivery among parturient women at a tertiary health institution in Yobe state.

## Methods

### Study site

This study was conducted from June to November 2014 at Federal Medical Center, a tertiary and referral health institution in Nguru, Yobe State, Nigeria. It is a 390-bed hospital and has an obstetrics and gynaecology department that provides antenatal care services. The antenatal clinic (ANC) runs 2 days per week (both for bookings and check-up), and the services rendered include health education, provision of IPTp-SP, distribution of ITNs, routine laboratory and radiological investigations. Nguru town is a malaria endemic area with transmission all the year round and peaks during the rainy season [3]. The town has the Nguru Lake that provides a breeding ground for the vector, mosquitoes. The total population of this area is 205,296 while the population of women of child-bearing age is 45,165 and that of pregnant women is 10,265. Majority of the women are housewives.

### Study design

Descriptive cross-sectional study design was used. The study population was parturient women delivering at FMC, Yobe. They must have been booked, had attended antenatal clinic in FMC, Nguru and presented in labour at term (between 36 and 40 weeks of gestation). Those with an eventful antenatal period, coexisting premorbid condition, preterm labour and who had no antenatal care and supporting records were excluded.

### Sample size

The study minimum sample size of 168 was calculated using sample size formula for single proportion with 12.5 % prevalence of malaria parasitaemia at delivery from a study in Sokoto [4], precision of 5 % and standard normal deviate of 1.96 at 95 % confidence intervals. In consideration of non-response rate of 10 %, the minimum sample needed for the study was 184.

### Patient’s recruitment and enrollment

Study participants were recruited consecutively as they presented during labour until the sample size was attained. Only those who had available supporting ANC record were included in the study. Written informed consent was obtained from each study participant or guardians for mothers less than 18 years of age before enrollment.

### Laboratory test for malaria parasite and anaemia

Trained laboratory scientists collected 5 mls of peripheral blood from the mother using vacutainer cup and needle and cord blood from the newborn into separate EDTA bottles from each study participant. Each sample was given a number and paired (mother-newborn) and labeled with the patient’s information. Thick and thin blood films were made from both venous and cord blood, then stained with Giemsa. They were examined microscopically using oil immersion objective (×100) for the presence of malaria parasite. Parasites were identified and densities estimated by counting against 200 leucocytes [5]. Slides without parasites were indicated as; No Malaria Parasites Seen (NMPS) while parasitaemia were graded as either low (1–999/high power field), moderate (1000–9999/high power field (HPF), or high (≥10,000/high power field). The stained slides were first read by the laboratory technician and secondly by a blinded reader (a WHO certified microscopist (level 1) with over 5 years of working experience) from the same institution. A third reader, a WHO microscopist (level 1 with more than years of working experience) from Kano State, read slides that had discordant readings by the first two readers. The packed cell volume (PCV) of the mothers was determined using capillary blood sample collected in a heparinized capillary tube. The samples were spun at 5000 rpm for 5 min in Microhaematocrit machine (Hawskley England) and the haematocrit reader was used to estimate the PCV. Presence of anaemia was considered as a PCV or haematocrit value less than 30 % [6, 7].

### Data collection and analysis

Trained research assistants administered a structured pre-tested questionnaire to collect information on demographics, malaria preventive strategies used, doses and trimester of administration of IPTp-SP and haematocrit values during the ANC visits and at delivery. Data on previous use of IPT, malaria infection and treatment during pregnancy were extracted from the ANC records. Digital weighing scale for babies was used in the labour ward by skilled midwives to measure the baby’s weight and recorded in the labour ward delivery register. Baby’s birth weight was collected from labour ward record. Low birth weight (LBW) babies were categorized as those with birth weight <2500 g [3].

Data collected for the study were entered, cleaned and analysed using Epi-info version 3.7 and Microsoft excel. Means, standard deviation and proportions were computed as relevant to summarize the data. Bivariate analysis (Chi square test) was used to determine association between categorical. Multiple logistic regression was carried out to determine predictors of malaria parasitaemia. Level of significance was set at 5 %.

### Ethical consideration

The ethical approval for the conduct of the study was obtained from the Institutional Ethics Review Committee of Federal Medical Center, Nguru. Written informed consent was obtained from each study participant or from guardians for mothers less than 18 years of age whose assents were also sought. Results of those found to have anaemia and parasitaemia were provided to the obstetricians for appropriate management of the patients. Information collected from the participants was kept confidential and stored in both hard-locked in cabinets and password- protected electronic files. Non-personal identifiers were used during analysis and presentation.

## Results

Overall, there were 268 women delivered at the health facility within the study period and 184 pregnant women in labour who met the inclusion criteria, were recruited and interviewed. The mean age of the respondents was 25.1 ± 2.5 years. More than half of them (54.0 %) were aged between 15 and 24 years, and the majority were primi- and secundigravidae (62.0 %).

Of the 184 parturient women, 74 (40.0 %) were positive for malaria parasites, 76 (41.0 %) had anaemia and 68 (37.0 %) had LBW babies (Table 1). Out of 74 parturient women who tested positive for malaria parasite at delivery, 24 (32 %) were detected using cord blood only while 5 (8 %) were detected using peripheral blood only. A total of 45 (61 %) also tested positive using both cord and peripheral blood samples. Of the 74 who had malaria parasitaemia at delivery, 39 (21.0 %) were aged between 15 and 24 years (Table 1). Of the 76 women who had anaemia, 46 (25.0 %) were in the 15–24 years age group, who were the majority (52.0 %) of the respondents (Table 1). Of the 68 parturient women, who had LBW babies, women in 12–29 years age group were the majority (37.0 %).

**Table 1:** Demographic characteristics and delivery outcome of parturient women at the Federal Medical Center, Nguru, Yobe State, June–November 2014

Characteristics	Frequency n (%)	MP+ n (%)	Anaemia^a^ n (%)	LBW n (%)
Age group (years)				
15–24	87 (40)	39 (21)	46 (25)	33 (18)
25–29	73 (47)	25 (14)	21 (11)	21 (11)
≥30	24 (13)	10 (5)	9 (5)	14 (8)
Total	184 (100)	74 (40)	76 (41)	68 (37)
Parity				
0–2	114 (62)	45 (24)	48 (27)	30 (16)
3–4	37 (20)	12 (7)	16 (9)	22 (12)
≥5	33 (18)	17 (9)	10 (5)	16 (9)
Total	184 (100)	74 (40)	76 (41)	68 (37)
Education level				
None	44 (24)	21 (11)	19 (10)	19 (10)
Primary	42 (23)	14 (8)	14 (8)	16 (9)
Secondary	74 (40)	31 (17)	38 (20)	26 (14)
Tertiary	24 (13)	8 (4)	5 (3)	7 (4)
Total	184 (100)	74 (40)	76 (41)	68 (37)
Monthly income (Naira)				
<5000	115 (62)	46 (25)	51 (27)	44 (24)
≥5000	69 (38)	28 (15)	25 (14)	24 (13)
Total	184 (100)	74 (40)	76 (41)	68 (37)

*MP+* participants tested positive for malaria parasite

*LBW* low birth (<2500 g)

^a^ Anaemia: packed cell volume <30 %

Only 80 (43.0 %) of the parturient women received up to two or more doses of IPTp-SP, while 57.0 % received only a dose or none. Majority of the women who had taken up to two doses were primi- and secundigravidae 45 (40.0 %), while only 35.0 % of the multigravidae had taken up to two doses. Almost all the respondents 173 (94 %) mentioned they slept under ITNs in the night preceding the interview.

Of 30 primi- and secundigravidae who delivered LBW babies, 19 (63 %) and 6 (20 %) had one and at least two doses of IPTp-SP respectively. Of 38 multigravidae who delivered LBW babies, 32 (84 %) and 4 (11 %) had one and at least two doses of IPTp-SP, respectively (Table 2).

**Table 2:** Association between dose of intermittent preventive treatment and low birth weight by gravidity among parturient women at Federal Medical Centre, Nguru, Yobe, Nigeria, June–November 2014

Dose of IPTp-SP received	LBW		Total n (%)
	Primi and secundigravidae n (%)	Multigravidae n (%)	
0	5 (17)	2 (5)	7 (10)
1	19 (63)	32 (84)	51 (75)
≥2	6 (20)	4 (11)	10 (15)
Total	30 (100)	38 (100)	68 (100)

On bivariate analysis, number of doses of SP (<2) used during pregnancy (OR: 3.1, 95 % CI: 1.5–6.7) presence of malarial infection at ANC enrollment (OR: 6.1, 95 % CI: 3.4–13.0) and method of IPT administration by prescription (OR 4.6, 95 % CI: 2.2–9.5) were significantly associated with presence of malaria parasitaemia among the parturient women (Table 3). The result of multivariate analysis showing predictors of maternal parasitaemia at delivery, indicated that those who had two doses of IPT were less likely to have parasitaemia (AOR: 0.4, 95 % CI: 0.2–0.8). In addition, the odds of parasitaemia among those prescribed SP and taken at home was 4 times more than that for those who had directly observed treatment (AOR 3.5, 95 % CI: 2.7–15.0). The odds of parasitaemia among those who had parasitaemia at time of booking was five times more (AOR 5.6, 95 % CI: 3.2–7.5) compared to those who did not (Table 4).

**Table 3:** Association between preventive strategies maternal malaria parasitaemia at delivery in Federal Medical Center Nguru, Yobe, Nigeria, June–November 2014

Variable/exposure factor	Maternal parasitaemia		Level of significance	
	Infected n = 74	Uninfected n = 110	OR (95 % CI)	*P* value
Doses of IPTp-SP received				
<2 doses (0,1)	63	78	3.1 (1.5–6.7)	<0.001
≥2 doses	11	42		
Method of IPTp-SP delivery				
Prescription	62	58	4.6 (2.2–9.5)	<0.001
DOT	12	52		
Malaria at ANC enrolment				
Positive	44	20	6.6 (3.4–13.0)	<0.001
Negative	30	90		
Use ITN				
Yes	68	97	1.5 (0.6–4.1)	0.31
No	6	13

**Table 4:** Determinants of malaria parasitaemia at delivery among parturient women in FMC, Yobe State

Term	AOR*	(95 % CI)″	*P* value^#^
IPTp-SP dose <2 ≥2	3.06	*1.8–5.2*	*<0.01*
Parity 0–2 >2	1.59	0.7–3.7	0.29
Monthly income ≥5000 <5000	0.93	0.4–2.1	0.88
Malaria Infection at ANC Yes No	5.58*	*3.2–7.5*	*<0.01*
Method of administration Prescription DOT	3.50*	*2.7–15.0*	*<0.01*

Italic values indicate significant associations

* AOR means adjusted odds ratio

″ 95 % confidence interval

^#^ *P* value significant at 0.05

## Discussion

Overall, 43 and 94 % of parturients received at least two doses of IPTp-SP and slept under ITN the night before the survey, respectively. The prevalence of malaria parasitaemia, anaemia and LBW was 40, 41 and 37 %, respectively among parturient women in this study. The 40 % malaria parasite prevalence among parturient women in this study was found to be quite high. Previous studies from Nigeria have reported different prevalence rates of parasitaemia at delivery ranging from 12.5 % in Sokoto [4] to as high as 69 % in Ekiti [8]. This wide variation may be due to the low IPT uptake and the intensity of the transmission. This study site is a high transmission zone and the study was conducted during the peak transmission season. Other reasons may be the irregular supply of sulfadoxine-pyrimethamine and non-adherence to directly observed treatment (DOT), which may be as a result of displacement from insurgence or the non-adherence of health workers to the administration of IPTp [3]. Parasitaemia was found to be higher among mothers less than 25 years of age in this study. This is consistent with the fact that pregnancy-associated malarial infection is more common in younger women, especially primigravidae, than in the older ones who have developed immunity from repeated exposures to malarial infection. This is similar to report from a multicentre study on malaria parasitaemia at parturition in Nigeria done in Ibadan, Kaduna, Ilorin and Enugu in 2009 [9] and another done in Sokoto [4] and other tertiary health institutions in Ilorin and Abuja on malaria infection at delivery in 2012 [10]. In this study, parasitaemia was also higher among those confirmed to have malaria during ANC and commenced on treatment than those who were presumably free of malarial infection at ANC booking enrolment. This may be related to how the pregnancy can affect the impact of malaria case management among pregnant women. The high prevalence of anaemia could be due to the high malaria prevalence, gravidity of the parturient women and possibly the income statue of the respondents. There was marked relationship between parasitaemia and anaemia. Maternal anaemia was found to be higher among women who had parasitaemia at delivery. The study also found relationship between anaemia and gravidity (primi and secundigravidae mostly affected) This is similar to findings of studies done in Ebonyi [11] and Benin [12] on the assessment of maternal anaemia in pregnancy and the effect of routine prevention, which reported a significant association between the presence of malaria infection at delivery and the presence of anaemia and also due to economic reason of sustenance (low income) [13]. Similar result was also found in Cameroon by Achidi et al. [14]. This finding is in contrast to study by Rijken et al. [15] in Asia, who found that malaria has no consistence relationship with anaemia. At booking only few of the patients (13 %) were anaemic while at delivery it was found to be high (41 %). This also explains the pregnancy period as a risk of developing anaemia may be due to infection, immune depression or even economic reason of sustenance. A similar result was reported in Benin in a study done by Huynh [16], who looked at the influence of timing of malaria infection during pregnancy on maternal anaemia and birth weight and found that infection in the early phase of pregnancy had a significant relationship with the presence of maternal anaemia at delivery.

The prevalence of low birth weight at delivery from this study was found to be 37 %. Parturient women who tested positive for malaria were mostly affected. There is relationship between low birth weight and gravidity and dose of IPTp-SP taken during pregnancy. Most of the low birth weight babies were born to mothers who have taken less than two doses of IPTp-SP and mostly multigravidae. This is similar to findings in the studies done by Gutman et al. in Malawi [17], Leport et al. in Benin [18] and Dennis et al. in Awka [19] who reported the association of prevention using IPT with SP at pregnancy, parasitaemia at delivery and low birth weight. Also a study with relationship between gravidity IPTp-SP dose and baby weight which reported that multigravidae were mostly affected was reported by Shulman et al. in Kenya [20].

Findings from this study has shown the relationship of parasitaemia at delivery and the effect of IPTp-SP dosage taken during pregnancy. It is evident from this study that the risk of developing parasitaemia at delivery upon administration of up to two doses of IPT was found to be protective compared to those who receive one or zero dose. This is similar to findings of an earlier work on effectiveness of IPTp-SP during pregnancy on maternal and birth outcome, reported in Malawi by Gutman et al. [17], whose findings suggested that there was a dose-dependent association on malarial infection at delivery. In addition, the method of IPT administration to the pregnant women during pregnancy was crucial to its effectiveness. It has been shown that the DOTS (directly observed treatment strategy) is a highly effective means of prevention than the mere prescription of the drugs for patients to buy and use them at their own convenience. The findings of a study done in Uganda by Mboya et al. [21] were similar and a systematic review by Boel et al. [6] advocated adherence to the guideline (DOTS) stipulated by WHO with regards to the method of IPTp-SP uptake during pregnancy.

In this study, using multivariate analysis, the independent significant predictors of the presence of malaria parasitaemia at delivery were the dosing effect of IPTp-SP (<2 doses), presence of malaria parasitaemia at ANC enrollment and not have IPT administered as DOT method. Similar studies that logically linked the presence of parasitaemia to various parameters were done in Benin [10, 15] where possible explanations included the resistance to the anti-malarial drug used during pregnancy, gravidity, number of antennal visits (dosage effect), maternal age and socioeconomic status.

Limitations of this study include the reliance on patients recall with regards to the administration of IPT through prescription (not DOT) and use of LLINs. Moreover, evidence of prescription from the patients records provides confirmation of IPT utilization, unlike those considered as LLIN users based on report of having slept under a LLIN the night before the study.

## Conclusions and recommendations

The effective use of malaria preventive strategy (IPTp-SP) was generally low and there was high prevalence of malaria parasitaemia, maternal anaemia and low weight babies. It is recommended that providing adequate information to pregnant women by health care providers can empower them with knowledge to demand for DOT mode of IPTp-SP. Also establishing a reliable mechanism by Government for ensuring adherence to DOT-based administration of appropriate dose for IPTp-SP is crucial.
